# A simple *SmR* selectable marker gene for Streptomycin/Spectinomycin selection in Arabidopsis nuclear transformation

**DOI:** 10.5511/plantbiotechnology.26.0420b

**Published:** 2026-06-25

**Authors:** Aoha Miki, Shunya Hirata, Juna Akiyama, Mana Shimatani, Kappei Kobayashi, Hidetaka Kaya

**Affiliations:** 1 Graduate School of Agriculture, Ehime University, Matsuyama, Ehime 790-8566, Japan; 2 Plant Molecular Biology and Virology, The United Graduate School of Agricultural Sciences, Ehime University, Matsuyama, Ehime 790-8566, Japan; 3 Faculty of Agriculture, Ehime University, Matsuyama, Ehime 790-8566, Japan

**Keywords:** *Arabidopsis thaliana*, AadA, SmR, Spectinomycin, Streptomycin

## Abstract

Selectable marker genes are essential for recovering rare transformants during plant genetic transformations. However, only a limited number of markers are routinely used in *Arabidopsis thaliana*, particularly for nuclear transformation by the floral dip method. Aminoglycoside-3″-adenylyltransferase (AadA) has been widely used in plastid transformation to confer resistance to Streptomycin and Spectinomycin; however, its applicability to nuclear transformation in Arabidopsis has not been systematically examined. Here, we evaluated an Arabidopsis-codon-optimized *AadA1* (*Streptomycin/Spectinomycin Resistance: SmR*) gene derived from *Escherichia coli* and lacking any plastid-targeting sequence, as a selectable marker for Arabidopsis nuclear transformation. Dose–response analyses revealed consistent Streptomycin and Spectinomycin sensitivity profiles across the four accessions (Col, L. *er*, Ws, and C24). When introduced *via* Agrobacterium-mediated floral dip, SmR conferred robust resistance to 50 mg l^−1^ Streptomycin or 10 mg l^−1^ Spectinomycin, enabling clear visual discrimination between resistant transformants and bleached non-transformants. Segregation analyses of T_2_ progeny revealed Mendelian 3 : 1 ratios, indicating successful transformation of the nuclear genome. Importantly, *SmR* transformants remained fully sensitive to Kanamycin and Hygromycin, demonstrating that SmR does not confer cross-resistance to these commonly used antibiotics. This compatibility enables the simultaneous use of Spectinomycin, Kanamycin, and Hygromycin for triple selection, allowing efficient isolation of triple transgenic plants. These results establish the non-targeted *SmR* as an efficient and cost-effective selectable marker for Arabidopsis nuclear transformation and expand the practical repertoire of plant selectable marker systems.

Selectable marker genes that confer resistance to antibiotics or herbicides are indispensable for identifying transformed cells during plant genetic transformation. In *Arabidopsis thaliana*, Kanamycin resistance (NPTII) and Hygromycin resistance (HPT) remain the most widely used nuclear markers, but additional markers are required to support multigene transformation and combinatorial reporter analyses.

Streptomycin and Spectinomycin are aminoglycoside antibiotics that inhibit bacterial translation by binding to the 30 S ribosomal subunit. Streptomycin induces mRNA misreading, whereas Spectinomycin binds to 16 S rRNA, blocking peptidyl-tRNA translocation (Wilson 2014). In plant cells, these antibiotics interfere with plastid translation, leading to the bleaching of sensitive tissues (Ahlert et al. 2003). Resistance to both antibiotics is mediated by Aminoglycoside-3″-adenylyltransferase (AadA), which inactivates Streptomycin and Spectinomycin through adenylylation (Flung et al. 1985). AadA-based cassettes have been widely employed in plastid genome transformation in tobacco (Svab and Maliga 1993), tomato (Wurbs et al. 2007), and Arabidopsis (Sikdar et al. 1998), where high levels of plastid-localized enzymes are required for efficient detoxification. However, their applicability to nuclear transformation has been explored far less. Recent study demonstrated that a stromal-targeted AadA construct can function as a nuclear selectable marker in several dicot species, including Arabidopsis (McCue et al. 2025). In this system, efficient selection requires fusion with a truncated stromal-targeting domain signal peptide derived from pea Rubisco small subunit (rbcS) and a sulfonamide resistance ORF (sul1) (pea STD trunc–sul1–AadA). To date, no study has evaluated whether a cytosolic, non-targeted AadA variant—specifically optimized for expression in Arabidopsis—is sufficient to confer Streptomycin or Spectinomycin resistance during nuclear transformation. Moreover, practical parameters such as accession-specific sensitivity thresholds, segregation behavior, and cross-resistance to other commonly used plant markers remain incompletely characterized. Addressing these knowledge gaps is essential for establishing robust and widely applicable aminoglycoside-based selection systems in Arabidopsis.

To determine the effective selection conditions, we examined Streptomycin and Spectinomycin sensitivity in four Arabidopsis accessions (Col, L. *er*, Ws, and C24). Seeds were germinated on 1/2 MS medium (half-strength Murashige and Skoog salts, half-strength Gamborg’s B5 vitamins, 1% sucrose, 0.5 g l^−1^ MES, 0.8% agar, pH 5.7–5.8) supplemented with 0–300 mg l^−1^ of each antibiotic and grown for three weeks at 21–22°C under long-day conditions (16-h light/8-h dark). In Col, chlorosis and growth arrest appeared at ≥30 mg l^−1^ Streptomycin, and complete bleaching occurred at ≥50 mg l^−1^ (Figure 1A). For Spectinomycin, severe bleaching occurred at ≥10 mg l^−1^. L. *er*, Ws, and C24 showed similar sensitivity profiles (Supplementary Figure S1). These concentrations (50 mg l^−1^ Streptomycin; 10 mg l^−1^ Spectinomycin) were therefore used for subsequent selection.

**Figure 1:**
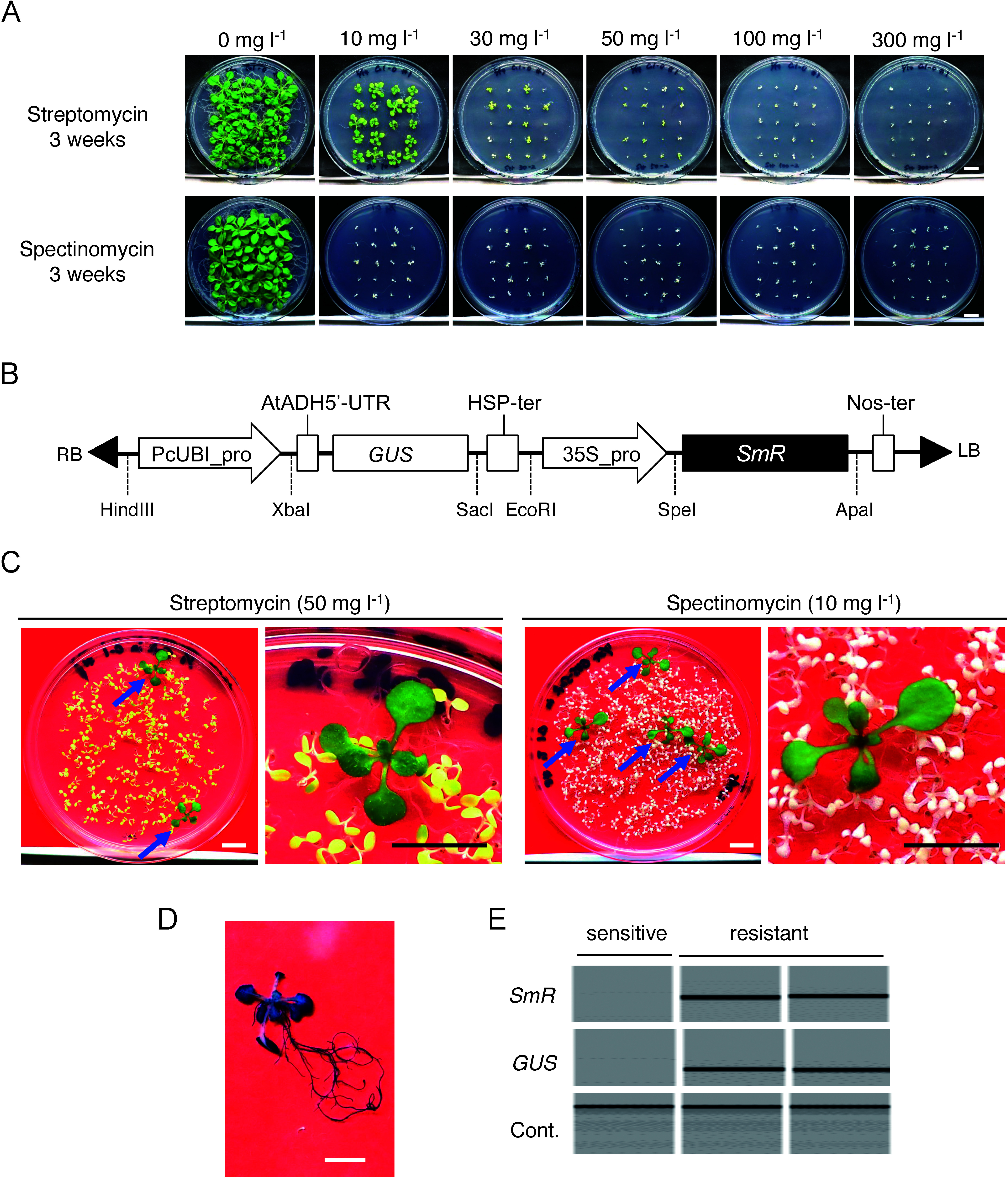
Figure 1. Selection of transgenic Arabidopsis using Streptomycin or Spectinomycin. (A) Dose-dependent sensitivity of Arabidopsis (Col) seedlings to Streptomycin and Spectinomycin. Scale bar indicates 1 cm. (B) Schematic representation of the T-DNA construct used for transformation. *SmR*, *Streptomycin/Spectinomycin resistance* gene; PcUBI, the promoter region of *Petroselinum crispum* *UBIQUITIN* gene; AtADH 5′-UTR, 5′-untranslated region of *Arabidopsis thaliana ALCOHOL DEHYDROGENASE* gene; *GUS*, β-glucuronidase reporter; HSP-ter, the terminator region of *Arabidopsis thalian*a *HEAT SHOCK PROTEIN 18.2 gene.* (C) T_1_ (Col) seedlings grown for approximately two weeks on 1/2 MS agar plates containing Streptomycin (50 mg l^−1^) or Spectinomycin (10 mg l^−1^). Arrows indicate resistant plants. Scale bars indicate 1 cm. (D) Histochemical GUS staining of Streptomycin-resistant T_1_ (Col) plants showing blue coloration in cotyledons and roots. Scale bar indicates 1 cm. (E) Genotyping of resistant and sensitive individuals among T_1_ (Col) plants. The *SmR* and *GUS* fragments were amplified by PCR, and a genomic region on chromosome 1 was used as a DNA control. DNA fragments were analyzed using the MultiNA MCE-202 DNA-500 system (Shimadzu, Japan).

We synthesized an Arabidopsis codon-optimized *AadA1* gene (*SmR*) based on the amino acid sequence of the *Escherichia coli* AadA1 protein (GenBank: ABB52514.1) (Lutz et al. 2006), which was predicted by WoLF PSORT (https://wolfpsort.hgc.jp/) to localize to the cytoplasm in plant cells. The *SmR* gene fragment was inserted at the SpeI–ApaI sites of pRI-PcUBI_pro::GUS_HPT (Kurokawa et al. 2021), replacing HPT, to generate pRI-PcUBI::GUS_SmR, driven by the CaMV 35S promoter (Figure 1B). Arabidopsis was transformed using Agrobacterium GV3130::pMP90 *via* the floral dip method.

T_1_ seeds derived from floral dip transformation were germinated on 1/2 MS medium containing 50 mg l^−1^ Streptomycin or 10 mg l^−1^ Spectinomycin, with 25 mg l^−1^ meropenem added to eliminate residual Agrobacterium. Under both conditions, a clear phenotypic distinction was observed: resistant seedlings displayed normal green cotyledons, whereas non-transformants exhibited bleaching and arrested growth (Figure 1C). Histochemical GUS staining was performed as previously described (Kaya et al. 2014) and revealed intense blue coloration in the cotyledons and roots of all resistant seedlings tested (Figure 1D). PCR genotyping was performed on resistant and sensitive T_1_ seedlings using primers specific to SmR, GUS, and an endogenous genomic control (Supplementary Table S1). Both *SmR* and *GUS* amplicons were detected in all resistant individuals, whereas neither fragment was amplified from the bleached seedlings (Figure 1E). These results demonstrate that resistance to Streptomycin or Spectinomycin directly reflects the presence of the T-DNA. Similar results were observed for L. *er*, Ws, and C24 (Supplementary Figure S3, S4).

To assess transgene segregation, T_2_ seeds from independently obtained T_1_ lines were germinated on Streptomycin- or Spectinomycin-containing medium. Both antibiotics yielded segregation patterns consistent with single-locus Mendelian inheritance. For example, one representative line displayed 114 resistant : 36 sensitive seedlings on Streptomycin medium, and 106 : 40 on Spectinomycin medium, both closely matching the expected 3 : 1 ratio (Table 1). These results indicate that the *SmR* gene can be used for transformant selection in the Arabidopsis nuclear genome. To test cross-resistance, T_2_ *SmR* transformants were germinated on 1/2 MS medium containing Kanamycin (25 mg l^−1^) or Hygromycin (10 mg l^−1^). All seedlings bleached and failed to develop, indicating complete sensitivity (Table 1). This demonstrates that *SmR* confers no detectable resistance to the antibiotics targeted by NPTII or HPT.

**Table 1:** Table 1. Segregation for antibiotics resistance in T2 progenies of SmR transgenic Arabidopsis (Col).

Antibiotics	Resistance	Sensitive	%R^a^	χ^2^
Streptomycin (50 mg l^−1^)	114	36	76.0	0.08^b^
Spectinomycin (10 mg l^−1^)	106	40	72.6	0.45^b^
Kanamycin (25 mg l^−1^)	0	144	0	—
Hygromycin (10 mg l^−1^)	0	148	0	—

%R^a^, percentage of antibiotics resistance seedlings; ^b^, no significantly different from 3 : 1 at *p*>0.05 (χ^2^=3.84).

In this study, we showed that the Streptomycin/Spectinomycin resistance gene *SmR*, derived from an Arabidopsis-codon-optimized *AadA1* variant, functions as an efficient selectable marker for nuclear transformation in *Arabidopsis thaliana*. Previous studies have suggested that efficient nuclear selection with AadA typically requires plastid targeting *via* stromal-targeting domain (McCue et al. 2025). In contrast, the AadA1 enzyme used in this study, which is predicted to localize to the cytosol and lacks any plastid-targeting sequence, provides robust resistance during nuclear transformation, demonstrating that fusion of a plastid-targeting signal to AadA is not required for nuclear genome transformation in *Arabidopsis thaliana*. This allows for a reduction in the size of the marker gene and simplifies its gene structure. The *SmR* marker enables clear visual identification of transformants based on green versus bleached phenotypes, confers reliable resistance to Streptomycin or Spectinomycin, and exhibits strict substrate specificity. While Streptomycin can also be used for selection, Spectinomycin generally provides clearer discrimination between resistant and non-transformed seedlings under our conditions. Because SmR transformants remain sensitive to Kanamycin and Hygromycin, this system is compatible with multigene transformation strategies that use multiple selectable markers (Supplementary Figure S5). Moreover, Streptomycin is inexpensive and widely available, reducing transformation costs compared with the Hygromycin-based system. Collectively, the *SmR* marker provides a practical, low-cost, and visually distinctive alternative to conventional NPTII and HPT systems.

## Abbreviations

AadA: Aminoglycoside-3″-adenylyltransferase
CaMV: Cauliflower mosaic virus
GUS: β-glucuronidase
HPT: Hygromycin phosphotransferase
NPTII: Neomycin phosphotransferase II
ORF: open reading frame
PcUBI: *Petroselinum crispum* UBIQUITIN
rbcS: Rubisco small subunit
SmR: Streptomycin/Spectinomycin Resistance
STD: stromal-targeting domain signal peptide
sul1: Sulfadiazine resistance gene

## Description of supplementary files

Supplementary Table S1. Primers used in this study.

Supplementary Figure S1. Dose-dependent sensitivity to Streptomycin or Spectinomycin in Arabidopsis accessions (L. *er*, Ws, and C24).

Supplementary Figure S2. Nucleotide sequence of the *SmR* gene.

Supplementary Figure S3. Screening of T_1_ transformants (L. *er*, Ws, and C24).

Supplementary Figure S4. GUS staining and genotyping of T-DNA of T_1_ transformants (L. *er*, Ws, and C24).

Supplementary Figure S5. Isolation and identification of triple transgenic plants (Col).
