# Supplementary material for: A simple SmR selectable marker gene for Streptomycin/Spectinomycin selection in Arabidopsis nuclear transformation
Source: Plant Biotechnol (Tokyo). 2026 Jun 25;43(2):233–6. doi: 10.5511/plantbiotechnology.26.0420b (PMC13324387; doi:10.5511/plantbiotechnology.26.0420b)
Supplement: Supplementary Data [file plantbiotechnology-43-2-26.0420b-s001.pdf]

Supplementary Table S1. List of primers used for genotyping

Primer name	Primer sequence
SmR_F1	5'-TACTAGAAGGGCTCTTATCAACGAC-3'
SmR_R1	5'-CCCTAGAAGCAAGTCTATCCTCTTC-3'
GUS-375	5'-GAAAAGTGTACGTATCACCGTTTGT-3'
GUS-879	5'-AAAGCCAGTAAAGTAGAACGGTTTG-3'
Cont. F1	5'-GACAACAAAACCCTGTTGTTTCTGAGC-3'
Cont. R1	5'-GACAACAAAACCCTGTTGTTTCTGAGC-3'
HYG-F10	5'-AGGAATCGGTCAATACACTACATGG-3'
HYG-R10	5'-TTTCCACTATCGGCGAGTACTTCTA-3'
NPTII-136F	5'-GTTCTTTTTGTCAAGACCGACCTG-3'
NPTII-629R	5'-CCACAGTCGATGAATCCAGAAAAG-3'

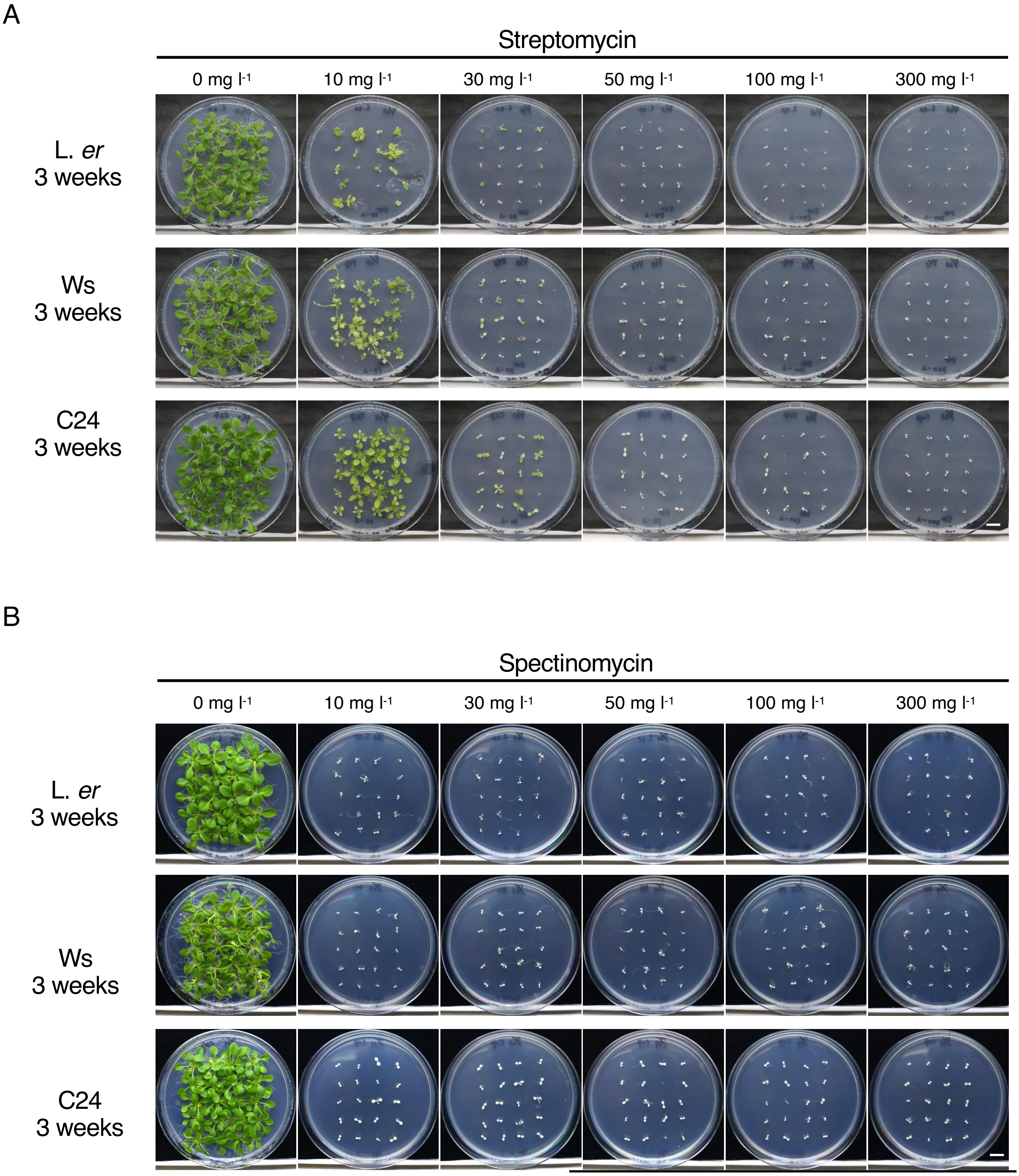

Supplementary Figure S1.

Dose-dependent sensitivity to Streptomycin or Spectinomycin in Arabidopsis accessions (L. *er*, Ws, and C24). Scale bar = 1 cm.

GTCCCCAGATTAGCCTTTTCAATTTCAGAAAGAATGCTAACCCACAGATGGTTAGAGAGGCTTACGCAGCAGGTCTCATCAAGACGATCTACCCG
AGCAATAATCTCCAGGAAATCAAATACCTTCCCAAGAAGGTTAAAGATGCAGTCAAAAGATTCAGGACTAACTGCATCAAGAACACAGAGAAAGA
TATATTTCTCAAGATCAGAAGTACTATTCCAGTATGGACGATTCAAGGCTTGCTTCACAAACCAAGGCAAGTAATAGAGATTGGAGTCTCTAAAA
AGGTAGTTCCCACTGAATCAAAGGCCATGGAGTCAAAGATTCAAATAGAGGACCTAACAGAACTCGCCGTAAAGACTGGCGAACAGTTCATACAG
AGTCTCTTACGACTCAATGACAAGAAGAAAATCTTCGTCAACATGGTGGAGCACGACACACTTGTCTACTCCAAAAATATCAAAGATACAGTCTC
AGAAGACCAAAGGGCAATTGAGACTTTTCAACAAAGGGTAATATCCGGAAACCTCCTCGGATTCCATTGCCCAGCTATCTGTCACTTTATTGTGA
AGATAGTGGAAAAGGAAGGTGGCTCCTACAAATGCCATCATTGCGATAAAGGAAAGGCCATCGTTGAAGATGCCTCTGCCGACAGTGGTCCCAAA
GATGGACCCCCACCCACGAGGAGCATCGTGGAAAAAGAAGACGTTCCAACCACGTCTTCAAAGCAAGTGGATTGATGTGATATCTCCACTGACGT
AAGGGATGACGCACAATCCCACTATCCTTCGCAAGACCCTTCCTCTATATAAGGAAGTTCATTTCATTTGGAGAGAACACGGGGGAACTAGT**ATG**
SpeI
GGAGAAGCTGTTATCGCTGAGGTGTCAACCCAGCTTTCTGAGGTTGTGGGAGTGATCGAGAGACACCTTGAGCCTACTCTTCTCGCTGTTCACCT
TTACGGATCTGCTGTTGATGGTGGACTCAAGCCGCACTCTGATATTGATCTTCTCGTGACTGTGACCGTGAGGCTCGATGAGACTACTAGAAGGG
CTCTTATCAACGACCTCCTCGAAACTTCTGCTTCTCCTGGTGAGTCTGAGATCCTCAGAGCTGTTGAGGTTACCATCGTGGTGCACGATGACATT
ATCCCTTGGCGTTACCCTGCTAAGCGTGAGCTTCAATTTGGAGAGTGGCAGAGGAACGACATCCTCGCTGGAATTTTTGAGCCTGCCACTATTGA
TATTGACCTCGCCATCCTCCTCACCAAGGCTAGAGAACATTCTGTTGCTCTCGTTGGACCTGCTGCTGAGGAACTTTTTGATCCTGTTCCTGAGC
AGGACCTCTTCGAGGCTCTTAACGAGACTCTTACCCTCTGGAACTCTCCTCCTGATTGGGCTGGTGATGAGAGAAACGTTGTGCTCACTCTCAGC
AGGATCTGGTACTCTGCTGTGACTGGAAAGATCGCTCCTAAGGATGTTGCTGCTGACTGGGCTATGGAAAGACTTCCTGCTCAATACCAGCCGGT
GATCCTTGAAGCTAGACAGGCTTATCTCGGGCAAGAAGAGGATAGACTTGCTTCTAGGGCTGACCAGCTCGAAGAGTTCGTTCATTACGTGAAGG
GCGAGATCACTAAGGTGGTGGGAAAGTGAGGGCCCCGATCGTTCAAACATTTGGCAATAAAGTTTCTTAAGATTGAATCCTGTTGCCGGTCTTGC
ApaI
GATGATTATCATATAATTTCTGTTGAATTACGTTAAGCATGTAATAATTAACATGTAATGCATGACGTTATTTATGAGATGGGTTTTTATGATTA
GAGTCCCGCAATTATACATTTAATACGCGATAGAAAACAAAATATAGCGCGCAAACTAGGATAAATTATCGCGCGCGGTGTCATCTATGTTACTA
GATC

Supplementary Figure S2.

Nucleotide sequence of the *SmR* gene.

Red: CaMV 35S promoter
Blue: *SmR* cds
Green: Nos terminator

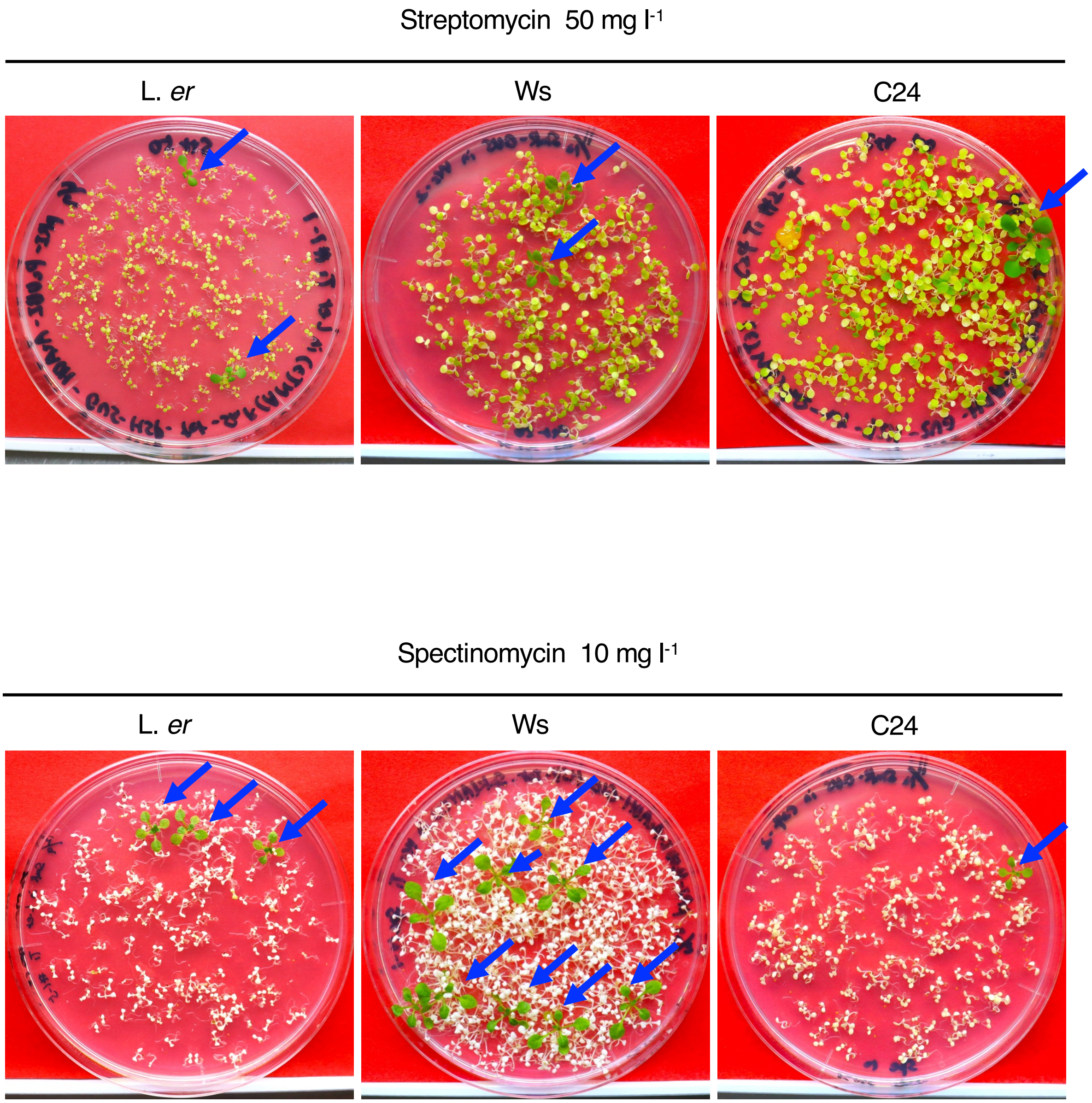

Supplementary Figure S3.

Screening of T_1 transformants (L. er, Ws, and C24). T_1 seeds screened on 1/2 MS agar plates containing Streptomycin (50 mg l^{-1}) or Spectinomycin (10 mg l^{-1}). T_1 seedlings were cultured for approximately two weeks under long-day conditions. Arrows indicate candidate transgenic plant.

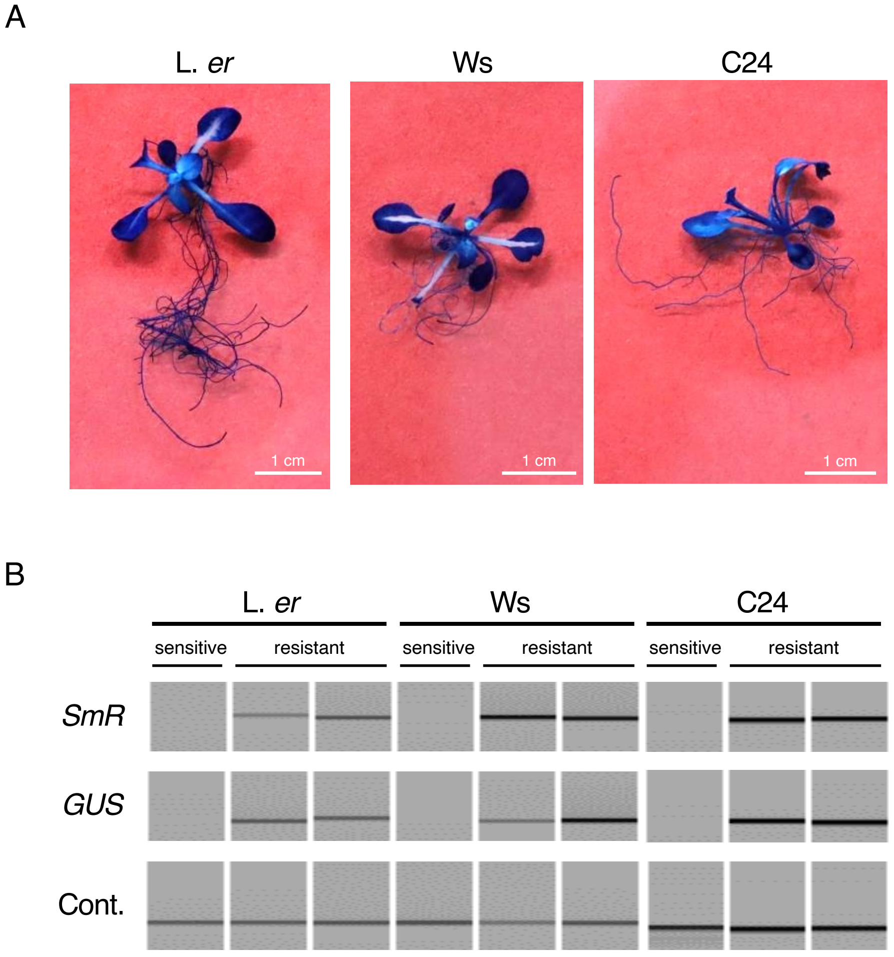

Supplementary Figure S4.

GUS staining and genotyping for T-DNA of T_1 transformants (L. er, Ws, and C24).

(A) Histochemical GUS staining of Streptomycin-resistant T_1 (L. *er*, Ws, and C24) plants.

(B) Genotyping of resistant and sensitive individuals from T_1 plants using specific primers for *SmR* and *GUS* genes. A genomic region on chromosome 1 was amplified as an internal DNA control. PCR products were analyzed using the MultiNA MCE-202 DNA-500 system (Shimadzu, Japan).

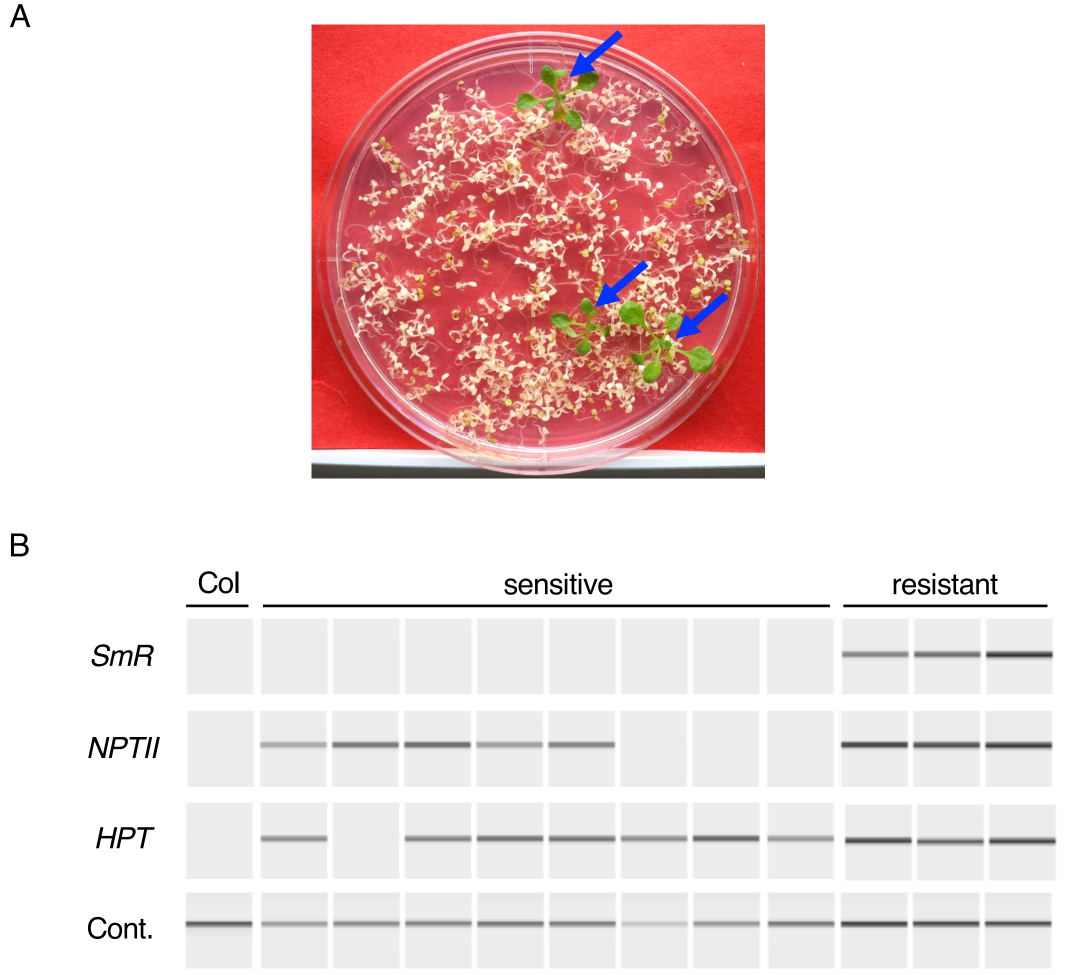

Supplementary Figure S5.

Isolation and identification of triple transgenic plants (Col).

(A) Double transgenic plants harboring the *NPTII* and *HPT* selectable marker genes were transformed with *Agrobacterium* carrying a T-DNA containing the *SmR* gene. T_1 seeds were screened on 1/2 MS agar plates containing Kanamycin (25 mg l^{-1}), Hygromycin (10 mg l^{-1}) , and Spectinomycin (10 mg l^{-1}). T_1 seedlings were cultured for approximately two weeks under long-day conditions. Arrows indicate candidate triple transgenic plants.

(B) Genotyping of triple transgenic plants. Genomic DNA was extracted from plants that were either resistant or sensitive to all three antibiotics, and the *SmR, NPTII*, and *HPT* genes were amplified by PCR. A genomic region on chromosome 1 was amplified as an internal DNA control. PCR products were analyzed using the MultiNA MCE-202 DNA-500 system (Shimadzu, Japan).
